# Risk factors for Fontan-associated hepatocellular carcinoma

**DOI:** 10.1371/journal.pone.0270230

**Published:** 2022-06-17

**Authors:** Tomomi Kogiso, Takaomi Sagawa, Makiko Taniai, Eriko Shimada, Kei Inai, Tokuko Shinohara, Katsutoshi Tokushige

**Affiliations:** 1 Department of Internal Medicine, Institute of Gastroenterology, Tokyo Women’s Medical University, Shinjuku-ku, Tokyo, Japan; 2 Pediatric Cardiology and Adult Congenital Cardiology, Tokyo Women’s Medical University, Shinjuku-ku, Tokyo, Japan; Peking Union Medical College, CHINA

## Abstract

**Aims:**

The incidence of hepatocellular carcinoma (HCC) in patients with Fontan-associated liver disease (i.e., FALD-HCC) has increased over time. However, the risk factors for HCC development remain unclear. Here, we compared the levels of non-invasive markers to the survival rate of FALD-HCC patients.

**Methods:**

From 2003 to 2021, 154 patients (66 men, 42.9%) developed liver disease after undergoing Fontan procedures. HCC was diagnosed in 15 (9.7%) (8 men, 53.3%) at a median age of 34 years (range, 21–45 years). We compared FALD-HCC and non-HCC cases; we generated marker level cutoffs using receiver operating characteristic curves. We sought to identify risk factors for HCC and mortality.

**Results:**

The incidence of HCC was 4.9% in FALD patients within 20 years after the Fontan procedure. Compared with non-HCC patients, FALD-HCC patients exhibited higher incidences of polysplenia and esophageal varices. At the time of HCC development, the hyaluronic acid (HA) level (p = 0.04) and the fibrosis-4 index (p = 0.02) were significantly higher in FALD-HCC patients than in non-HCC patients; the total bilirubin (T-BIL) level (p = 0.07) and the model for end-stage liver disease score [excluding the international normalized ratio (MELD-XI)] (p = 0.06) tended to be higher in FALD-HCC patients. Within approximately 20 years of the Fontan procedure, 10 patients died (survival rate, 96.9%). Kaplan–Meier curve analysis indicated that patients with T-BIL levels ≥ 2.2 mg/dL, HA levels ≥ 55.5 ng/mL, and MELD-XI scores ≥ 18.7 were at high risk of HCC, a generally poor prognosis, and both polysplenia and esophageal varices. Multivariate Cox regression analyses indicated that the complication of polysplenia [Hazard ratio (HR): 10.915] and a higher MELD-XI score (HR: 1.148, both p < 0.01) were independent risk factors for FALD-HCC.

**Conclusions:**

The complication of polysplenia and a MELD-XI score may predict HCC development and mortality in FALD patients.

## Introduction

The Fontan procedure is a palliative operation for patients with single-ventricle congenital heart disease [1]. Postoperatively, the superior vena cava drains into the distal right pulmonary artery (the “Fontan circulation”) [1]. Systemic venous hypertension developing secondary to establishment of the Fontan circulation decreases the venous return, in turn reducing the cardiac output, which increases cardiac pressure and dilates the sinusoid. Fontan-associated liver disease (FALD) may thus develop, particularly in patients with long-term hepatic venous congestion and hypoxia [2]. A histological analysis found that 43% of patients showed advanced liver fibrosis at 30 years after the Fontan procedure [3]. Furthermore, benign tumors [focal nodular hyperplasia (FNH) and hepatic adenoma] and hepatocellular carcinoma (HCC) have been reported after the Fontan procedure [4–6]. In one national survey [7], liver cirrhosis or HCC was reported in 31/2,700 cases (1.15%). We previously described the characteristics of HCC in patients with FALD (i.e., FALD-HCC) [8]; the respective incidences were 0.8% and 2.9% at 10 and 20 years after the Fontan procedure. The liver nodules are usually hyperechoic, hypervascular, and predominantly peripheral [9]. Nii et al. [10] reported that a high central venous pressure (CVP) [hazard ratio (HR), 1.1; 95% confidence interval (CI), 1.003–1.2; p = 0.04] and severe atrioventricular valve regurgitation (hazard ratio, 6.0; 95% confidence interval, 1.5–24; p = 0.01) evident in the early postoperative period were independently predictive of liver cirrhosis and HCC.

In terms of non-invasive markers of FALD fibrosis, the levels of hyaluronic acid (HA) and type IV collagen 7S are typically increased in FALD patients [11, 12]. Moreover, a higher aspartate aminotransferase-to-platelet ratio index (APRI) and an elevated fibrosis-4 (FIB-4) index reflected greater fibrosis in FALD patients [8, 13]. Although the model for end-stage liver disease (MELD) score is used to estimate the prognosis of patients with liver disease, the score cannot be used for FALD patients because they are treated with warfarin. Therefore, the MELD score excluding the international normalized ratio (i.e., the MELD-XI score) is generally used to evaluate such patients [14]. Here, we studied a greater number of FALD and HCC cases than in any previous study; we defined risk factors for HCC and mortality by reference to the cutoffs of several non-invasive markers.

## Methods

### Patients and study design

This single-center retrospective study enrolled 154 FALD patients who had been referred to our department between 2003 and 2020 for liver damage or space-occupying lesions of the liver. We collected detailed clinical and demographic information regarding those patients. Laboratory data were obtained from the time of presentation with FALD or HCC. FALD was diagnosed on the basis of abnormalities in liver structure, as well as elevated liver enzyme or bilirubin levels attributable to the Fontan circulation [15]. We compared patients who did and did not develop FALD-HCC, as well as patients who died and patients who survived. Markers that were significant in terms of predicting between-group differences were identified. We derived cutoffs for all markers by constructing receiver operating characteristic (ROC) curves. We used the cutoffs for comparisons between groups. The study protocol adhered to the principles of the Declaration of Helsinki and the ethical guidelines of the Tokyo Women’s Medical University Hospital (Tokyo, Japan). The Institutional Review Board of the Tokyo Women’s Medical University Hospital approved the study protocol. Informed consent was obtained from all participants.

### Clinical parameters

The following baseline characteristics were assessed: age, sex, date of the Fontan procedure, congenital complications, and the use of warfarin potassium. The following laboratory parameters were measured at the first visit to our department and again at the time of HCC development: serum levels of albumin (g/dL), total bilirubin (T-BIL, mg/dL), aspartate aminotransferase (U/L), alanine transaminase (U/L), and gamma-glutamyl transferase (U/L); platelet count (× 10^4^/μL); levels of brain natriuretic peptide (BNP, pg/mL) and alpha-fetoprotein (ng/mL); and positivity status in terms of hepatitis B surface antigen and anti-hepatitis C virus antibody. The MELD-XI score [14] and the Child-Turcotte-Pugh (CTP) score [16] were used to evaluate liver function. Patients with protein-losing enteropathy (PLE) were excluded at the time of calculation of the CTP score. In patients prescribed warfarin potassium, the prothrombin % was set to 1 point of the CTP score. To evaluate fibrosis, the levels of HA (ng/mL), type IV collagen 7S (ng/mL), and type III procollagen-N-peptide (P-III-P, ng/mL) were assayed; the FIB-4 index and APRI score were calculated, as described previously [17, 18]. P-III-P levels measured by radioimmunoassay (U/mL) were converted to the units (ng/mL) of the chemiluminescent immunoassay method as follows: 11.773 x (radioimmunoassay value) + 7.175 ng/mL.

### Diagnosis of HCC

HCC was diagnosed histologically or based on abdominal ultrasound, abdominal enhanced computed tomography, and/or gadolinium ethoxybenzyl diethylenetriamine penta-acetic acid (Bayer Schering Pharma, Berlin, Germany)-enhanced magnetic resonance imaging; and/or an elevated alpha fetoprotein level [19].

### Endoscopic diagnosis of esophageal varices

Endoscopic diagnosis of esophageal varices was based on the Japanese Research Society for Portal Hypertension guidelines [20]. The timing of upper endoscopy varied, and the results of the latest examination were used.

### Cardiac catheterization

Cardiac catheterization was performed using a routine procedure. The central venous pressure (CVP, mmHg) and ejection fraction (EF, %) were monitored. Cardiac catheterization was performed before, at the time of, and after the operation, or when the symptoms of cardiac failure appeared. The data from the latest visit or from the time of HCC diagnosis were used.

### Statistical analysis

Data are presented as medians with ranges. We compared data between patients with and without FALD-HCC using the Mann–Whitney U test and the χ^2^ test in SPSS software (version 25.0; IBM Corp., Armonk, NY, USA). Differences were considered statistically significant at p < 0.05. HCC development after the Fontan procedure was evaluated by constructing Kaplan–Meier curves. The survival rate according to FALD-HCC status was estimated using the log-rank test. The diagnostic performances of markers, the levels of which significantly differed between groups, were assessed via ROC curve analysis. The probabilities of true-positive (sensitivity) and true-negative (specificity) assessments were determined at selected cutoffs, and the areas under the ROC curves (AUROCs) were calculated.

Univariate and multivariate Cox regression analyses were used to evaluate the risk for HCC. HRs and 95% CIs were calculated. The following factors were analyzed: age, polysplenia, esophageal varices, HA, FIB-4 index, and the MELD-XI scores.

## Results

### Characteristics of patients with FALD and FALD-HCC

The Fontan procedure was performed at a median age of 5.7 years (range, 0.0–37.5 years, Table 1). Patients developed FALD at a median age of 26.2 (range, 6.4–51.0) years. Hyperechoic lesions were seen in 95 cases, while 26 space-occupying lesions with diameters ≥ 10 mm were suspected to be FNHs based on the hyper-vascularity on CT scans. Two cases were pathologically diagnosed as FNHs. HCC was diagnosed in 15 cases (8 men, 53.3%) at a median age of 34.4 years (range, 20.6–45.4 years); the median interval between the Fontan procedure and HCC diagnosis was 26.1 years (range, 14.9–31.2 years). Six cases of HCCs were diagnosed based on dynamic CT scans and tumor makers. In two cases, a diagnosis of HCC was made on the basis of CT findings and tumor growth over time, or angiography. The remaining seven cases were diagnosed pathologically. The most common cardiac diseases were tricuspid valve insufficiency (n = 5) and a double-outlet right ventricle (n = 6). Polysplenia (n = 3) and esophageal varices (n = 5) were frequently observed in FALD-HCC patients; many such cases were also hepatitis C virus-positive (p = 0.02).

**Table 1:** Characteristics of FALD patients.

	Total (n = 154)	Non-HCC (n = 139)	HCC (n = 15)	p-value (non-HCC vs. HCC)
Age at the time of diagnosis of FALD or HCC (years)	26.2 (6.4–51.0)	25.7 (6.4–51.0)	34.4 (20.6–45.4)	0.01
Age at the time of Fontan operation (years)	5.7 (0.0–37.5)	5.5 (0.0–37.5)	6.2 (4.0–25.6)	0.98
Men (%)	66 (42.9%)	58 (41.7%)	8 (53.3%)	0.39
**Cardiac disease (%)**				0.18
Single cardiac ventricle	46 (29.9%)	45 (32.4%)	1 (6.7%)	
Pulmonary atresia	12 (7.8%)	10 (7.2%)	2 (13.3%)	
Tricuspid valve insufficiency	37 (24.0%)	32 (23.0%)	5 (33.3%)	
Double-outlet right ventricle	44 (38.6%)	38 (27.3%)	6 (40.0%)	
**Complications (%)**				0.05
Asplenia	18 (11.7%)	18 (12.9%)	0 (0.0%)	
Polysplenia	12 (7.8%)	9 (6.5%)	3 (20.0%)	
Visceral inversions	14 (9.1%)	9 (6.5%)	5 (33.3%)	
PLE	5 (3.2%)	3 (2.2%)	2 (13.3%)	
HBs antigen-positive	1 (0.6%)	1 (0.7%)	0 (0.0%)	0.74
HCV antibody-positive	5 (3.2%)	3 (2.2%)	2 (13.3%)	0.02
**Ultrasonic cardiography (n = 133)**				
Aortic valve regurgitation (%)	94 (70.7%)	86 (71.1%)	8 (66.7%)	0.54
Tricuspid regurgitation (%)	33 (24.8%)	31 (25.6%)	2 (16.7%)	0.44
Inferior vena cava (mm)	17.3 (5.5–34.0)	17.1 (5.5–34.0)	18.1 (16.0–22.0)	0.40
Systolic fraction	0.27 (0.10–0.59)	0.27 (0.10–0.59)	0.28 (0.19–0.39)	0.50
**Cardiac catheterization (n = 142)**				
CVP (mmHg)	12 (4–31)	12 (4–31)	11 (9–18)	0.67
EF (%)	52 (22–86)	52 (22–86)	56 (41–62)	0.43

FALD, Fontan-associated liver disease; HCC, hepatocellular carcinoma; PLE, protein-losing enteropathy; HBs antigen, hepatitis B surface antigen; HCV, hepatitis C virus; CVP, central venous pressure; EF, ejection fraction.

In terms of laboratory data at the first visit to our department (Table 2), the T-BIL level (FALD-HCC vs. non-HCC patients, 1.9 vs. 1.2 mg/dL, p = 0.04), FIB-4 index (1.81 vs. 0.72, p = 0.03), and MELD-XI score (18.17 vs. 11.58, p = 0.01) were significantly higher in FALD-HCC patients than in non-HCC patients; the HA level (67.0 vs. 42.5 ng/mL, p = 0.08) and the CTP score (6 vs. 6, p = 0.05) tended to be higher in FALD-HCC patients. At the time of HCC diagnosis, the HA level (93.0 vs. 42.5 ng/mL, p = 0.04) and the FIB-4 index (1.80 vs. 0.82, p = 0.02) were significantly higher in FALD-HCC patients than in non-HCC patients. The T-BIL level (2.0 vs. 1.2 mg/dL, p = 0.07) and the MELD-XI score (15.68 vs. 11.58, p = 0.06) tended to be higher in FALD-HCC patients than in non-HCC patients. The values did not significantly differ between the first visit and the time of HCC diagnosis. Seventy-one patients were on warfarin during the study.

**Table 2:** Laboratory data on FALD patients.

	Total (n = 154)	Non-HCC (n = 139)	HCC (n = 15) at the first visit	p-value; non-HCC vs. HCC patients at the first visit	HCC (n = 15)	p-value (non-HCC vs. HCC patients)
Age (years)	26.2 (6.4–51.0)	25.7 (6.4–51.0)	32.5 (18.5–45.4)	0.01	34.4 (20.6–45.4)	0.01
Albumin (g/dL)	4.5 (2.6–5.7)	4.5 (2.6–5.7)	4.2 (2.4–5.3)	0.05	4.3 (3.3–5.3)	0.28
Total bilirubin (mg/dL)	1.2 (0.3–7.2)	1.2 (0.3–7.2)	1.9 (0.6–3.7)	0.04	2.0 (0.6–3.1)	0.07
Aspartate aminotransferase (U/L)	24 (10–138)	24 (10–138)	24 (15–216)	0.29	27 (15–91)	0.38
Alanine transaminase (U/L)	22 (4–241)	21 (4–241)	22 (6–96)	0.90	25 (6–40)	0.26
Gamma-glutamyl transferase (U/L)	82 (17–876)	79 (17–876)	130 (33–454)	0.19	115 (32–331)	0.40
PT% *	70.1 (19.7–100.0)	70.1 (19.7–100.0)	71.3 (53.0–78.7)	0.66	71.3 (53.0–78.7)	0.77
PT-INR *	1.15 (1.86–3.24)	1.15 (1.86–3.24)	1.15 (1.06–1.32)	0.48	1.15 (1.09–1.32)	0.66
Platelet count (×10^4^/μL)	15.8 (4.6–50.1)	15.9 (4.6–50.1)	11.3 (5.9–24.3)	0.13	12.4 (5.7–24.3)	0.14
BNP (pg/mL)	66.8 (7.7–1,138.4)	66.8 (7.7–751.1)	135.7 (24.0–1,138.4)	0.33	76.8 (17.9–1,138.4)	0.45
Alpha-fetoprotein (ng/mL)	4 (1–81,663)	4 (1–10,896)	8 (4–81,663)	0.19	7 (3–81,663)	0.24
Hyaluronic acid (ng/mL)	44.0 (9.0–244.0)	42.5 (9.0–244.0)	67.0 (35.0–200.0)	0.08	93.0 (36.0–200.0)	0.04
Type IV collagen 7S (ng/mL)	7.9 (3.3–13.0)	7.8 (3.3–13.0)	8.8 (4.9–12.0)	0.19	8.5 (4.9–12.0)	0.45
P-III-P (ng/mL)	15.5 (6.5–53.1)	15.4 (6.5–53.1)	16.5 (13.3–22.5)	0.91	17.0 (14.3–22.5)	0.73
APRI	0.50 (0.13–2.44)	0.49 (0.13–1.75)	0.74 (0.25–2.70)	0.10	0.61 (0.22–2.44)	0.13
FIB-4 index	0.84 (0.15–6.30)	0.82 (0.15–6.30)	1.81 (0.53–4.59)	0.03	1.80 (0.53–4.59)	0.02
MELD-XI	11.58 (8.20–32.66)	11.58 (8.20–32.66)	18.17 (9.44–24.83)	0.01	15.68 (9.44–23.73)	0.06
CTP score **	6 (4–10)	6 (4–10)	6 (5–9)	0.05	5 (5–8)	0.25

FALD, Fontan-associated liver disease; HCC, hepatocellular carcinoma; PT, prothrombin time; INR, international normalized ratio; BNP, brain natriuretic peptide; P-III-P, type III procollagen-N-peptide; APRI, aspartate aminotransferase-to-platelet ratio index; FIB-4, fibrosis-4 index; CTP score, Child-Turcotte-Pugh score; MELD-XI, model for end-stage liver disease excluding the INR.

* Patients treated with warfarin potassium (n = 71) were excluded.

** Patients with PLE (n = 5) were excluded.

Ultrasonic cardiography results showed aortic valve regurgitation in eight cases (66.7%), tricuspid regurgitation in two cases (16.7%), inferior vena cava 18.1 mm (16.0–22.0 mm), and systolic fraction 0.28 (0.19–0.39) in 12 cases of HCC (Table 1). However, none of the factors had a statistically significant difference compared to non-HCC cases.

Cardiac function was monitored via cardiac catheterization (n = 142). The CVP was 12 (range, 4–31) mmHg and the EF was 51% (22–86%) in non-HCC cases. In patients with HCC, the CVP was 11 (9–18) mmHg and the EF was 56% (41–62%). These values did not significantly differ (p = 0.43).

**Fig 1:**
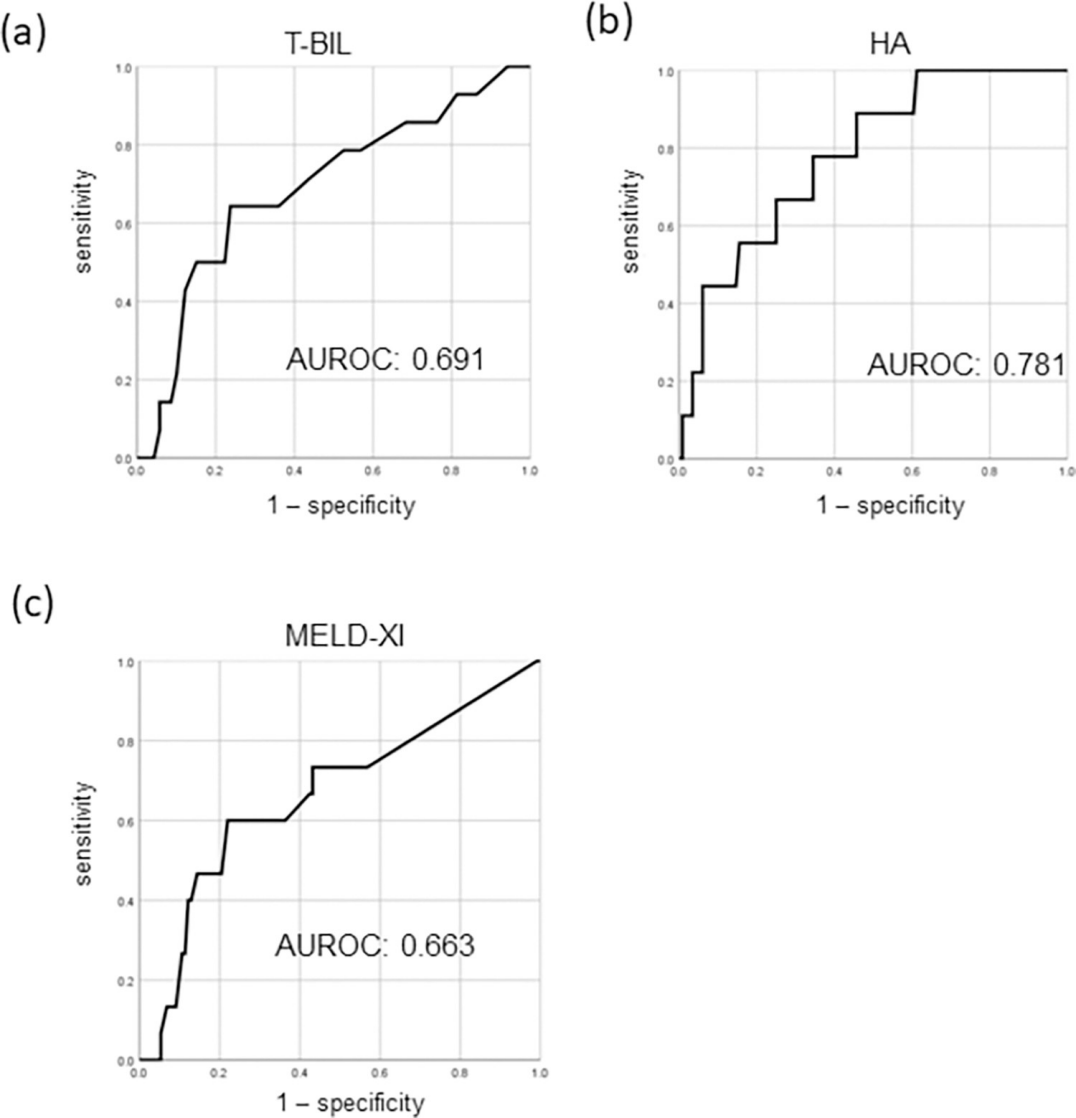
ROC curve data and FALD-HCC development. T-BIL level; b) HA level; and c) MELD-XI score. The T-BIL cutoff for HCC diagnosis based on the ROC curves was ≥ 2.2 mg/dL (sensitivity, 0.500; specificity, 0.849; AUROC, 0.691; a); for HA, the cutoff was ≥ 55.5 ng/ mL (sensitivity, 0.778; specificity, 0.612; AUROC, 0.781; b); for the MELD-XI score, the cutoff was ≥ 18.7 (sensitivity, 0.400; specificity, 0.871; AUROC, 0.663; c). AUROC, area under the ROC curve; HA, hyaluronic acid; HCC, hepatocellular carcinoma; MELD-XI, model for end-stage liver disease excluding the international normalized ratio; ROC, receiver operating characteristic; T-BIL, total bilirubin.

The cutoffs for HCC detection at the time of diagnosis as revealed by the ROC curves were T-BIL ≥ 2.2 mg/dL (sensitivity, 0.500; specificity, 0.849; AUROC, 0.691; Fig 1A); HA ≥ 55.5 ng/mL (sensitivity, 0.778; specificity, 0.612; AUROC, 0.781; Fig 1B); and MELD-XI score ≥ 18.7 (sensitivity, 0.400; specificity, 0.871; AUROC, 0.663; Fig 1C).

### Comparison of patients with and without HCC, and the HCC risks

**Fig 2:**
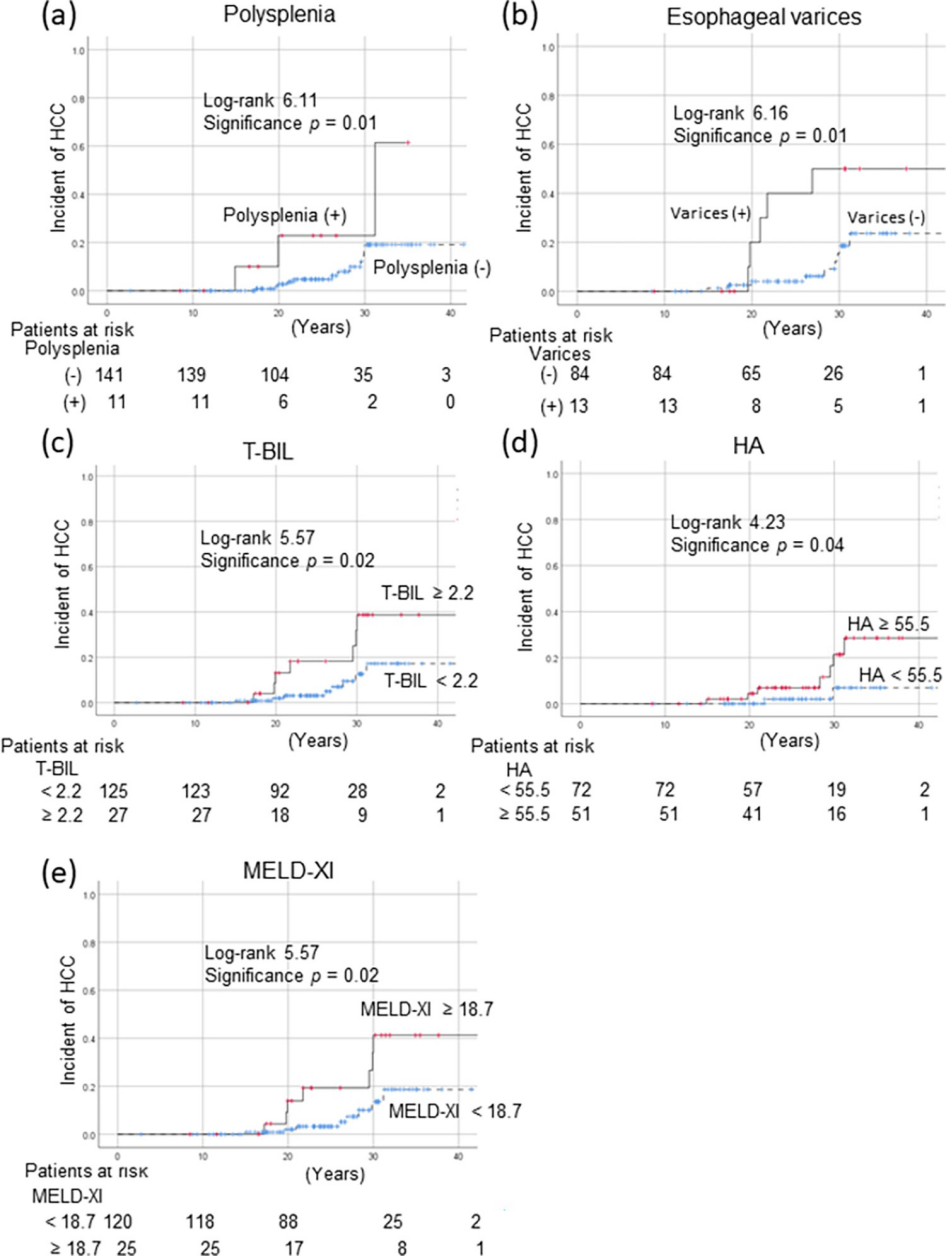
HCC incidences after the Fontan procedure in subgroups divided according to a) Polysplenia status, b) esophageal varices status, c) T-BIL level, d) HA level, and e) MELD-XI score. HCC incidences were calculated using the Kaplan–Meier method. Subgroup analyses showed that polysplenia and esophageal varices significantly increased the risk of HCC (both p = 0.01, panels a and b). Furthermore, in patients with T-BIL levels ≥ 2.2 mg/dL (p = 0.02, c), HA levels ≥ 55.5 ng/mL (p = 0.04, d), and MELD-XI scores ≥ 18.7 (p = 0.02, e), the HCC risk was significantly increased. HA, hyaluronic acid; HCC, hepatocellular carcinoma; MELD-XI, model for end-stage liver disease excluding the international normalized ratio; T-BIL, total bilirubin.

HCC incidences were calculated using the Kaplan–Meier method (Fig 2). These were 0.8%, 2.9%, and 13.3%, respectively, at 10, 20, and 30 years after the Fontan procedure in FALD patients. Subgroup analysis showed that both polysplenia and esophageal varices significantly increased the HCC risk (both p = 0.01, Fig 2A and 2B). The HCC incidence was compared using the cutoff values for HCC diagnosis as revealed by ROC analysis. In patients with T-BIL levels ≥ 2.2 mg/dL (p = 0.02, Fig 2C), HA levels ≥ 55.5 ng/mL (p = 0.04, Fig 2D), and MELD-XI scores ≥ 18.7 (p = 0.02, Fig 2E), the HCC risk was significantly increased.

### Survival rates of FALD patients by risk factors

**Fig 3:**
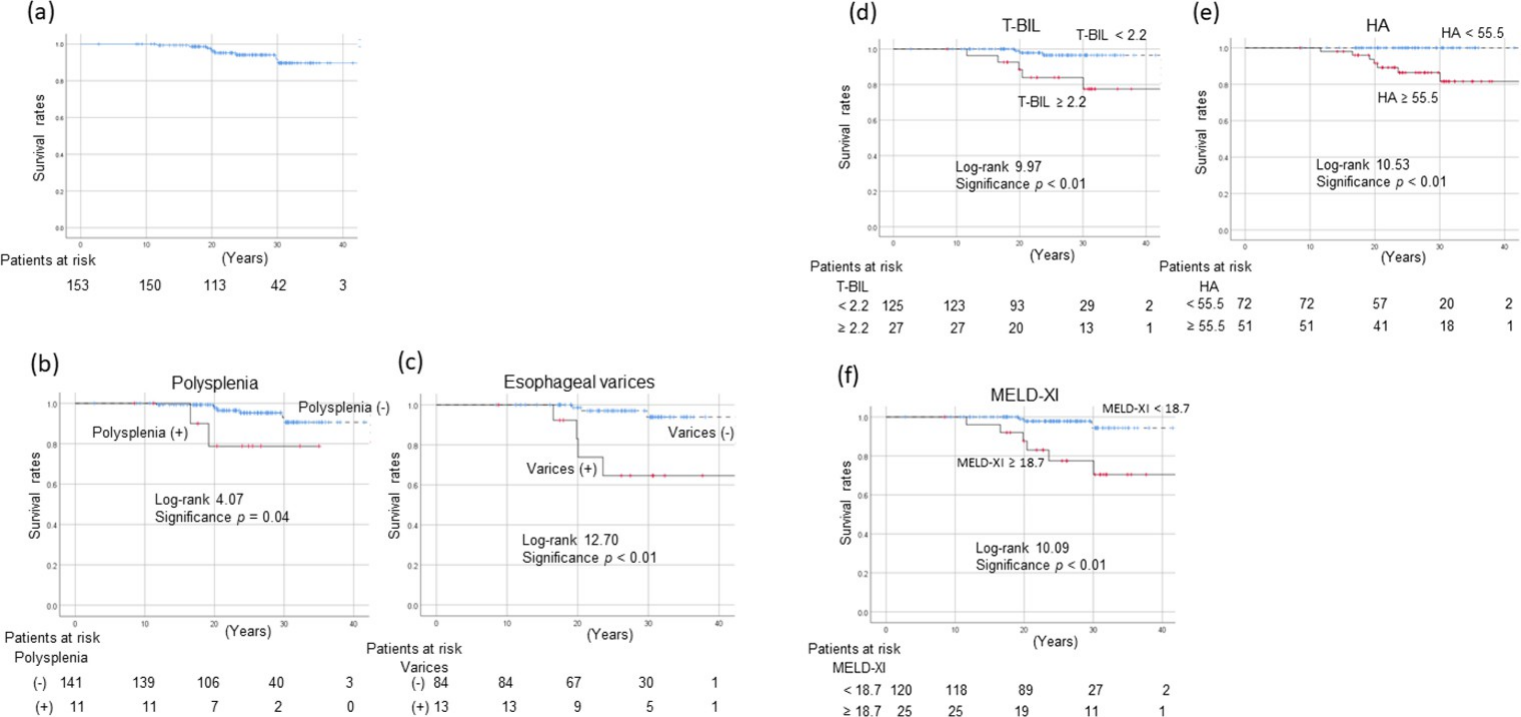
Survival rates after the fontan procedure stratified according to subgroup. a) Overall survival, and survival stratified according to b) polysplenia status; c) esophageal varices status; d) T-BIL level; e) HA level; and f) MELD-XI score at the time of HCC diagnosis. Survival rates were estimated using the Kaplan–Meier method. The survival rate was 96.9% at 20 years after surgery (a). Survival was poor in patients with polysplenia (p = 0.04, b) and esophageal varices (p < 0.01, c). Patients with T-BIL levels ≥ 2.2 mg/dL (p < 0.01, d), HA levels ≥ 55.5 ng/mL (p < 0.01, e), and MELD-XI scores ≥18.7 (p < 0.01, f) exhibited worse outcomes than did other patients. HA, hyaluronic acid; MELD-XI, model for end-stage liver disease excluding the international normalized ratio; T-BIL, total bilirubin.

Of the 154 FALD cases, 10 died with 20 years of the Fontan procedure (survival rate, 96.9%; Fig 3A). The causes of death were liver-related in six, sepsis in two, and “other” in two. The survival rate was poor in patients with polysplenia (p = 0.04, Fig 3B) and esophageal varices (p < 0.01, Fig 3C). Patients with T-BIL levels ≥ 2.2 mg/dL (p < 0.01, Fig 3D), HA levels ≥ 55.5 ng/mL (p < 0.01, Fig 3E), and MELD-XI scores ≥ 18.7 (p < 0.01, Fig 3F), exhibited worse survival than did other patients.

Multivariate Cox regression analyses were used to evaluate the risk for HCC using age, polysplenia, esophageal varices, HA, FIB-4 index, and MELD-XI scores. Polysplenia (HR: 10.915, 95% CI: 2.603–45.764, p < 0.01) and higher MELD-XI scores (HR: 1.148, 95% CI: 1.038–1.270, p < 0.01) were identified as risk factors for FALD-HCC.

## Discussion

We encountered 15 (9.7%) HCC cases among 154 FALD cases within approximately 20 years of the Fontan procedure. Fibrosis was associated with HCC development, as were polysplenia, esophageal varices, elevated T-BIL and HA levels, and higher MELD-XI scores. In particular, polysplenia and higher MELD-XI scores were independent risk factors for HCC development. The survival rate was 96.9% within 20 years after surgery; the abovementioned markers were predictive of mortality.

HCC can develop long after the Fontan procedure; liver cirrhosis is a major risk factor for FALD-HCC [21]. In one multicenter study [22], approximately half of all cases exhibited liver cirrhosis at the time of HCC diagnosis. Here, we found that HCC was more common in patients with esophageal varices (i.e., liver cirrhosis) than in other patients. Isoura et al. [23] reported that the platelet count and CT scans were useful in screening for esophageal varices. Therefore, gastrointestinal examination and HCC surveillance are appropriate for patients with low platelet counts.

De Bruyne et al. [24] reported that the aspartate aminotransferase, alanine transaminase, gamma-glutamyl transferase, and direct bilirubin levels were increased in 12 (34%), 5 (14%), 24 (69%), and 7 (20%) Fontan patients, respectively; the platelet count was decreased in 7 (20%) Fontan patients. Here, we found that the T-BIL level tended to be increased in FALD-HCC patients; it was a useful predictor of FALD-HCC. Notably, a T-BIL level > 2.2 mg/dL served as a marker of fibrosis progression and an increased risk of HCC. However, its sensitivity was low (0.500).

In terms of non-invasive fibrosis markers, the levels of HA and type IV collagen 7S [11, 12] typically increase if liver complications develop; these levels are useful when evaluating FALD status. In contrast, the P-III-P level is susceptible to inflammation status. Shimizu et al. [25] suggested that the HA and gamma-glutamyl transferase levels are useful for predicting liver fibrosis progression in Fontan patients. In this study, we found that the HA level was significantly increased in FALD-HCC patients, compared with other patients. An HA cutoff of 55.5 ng/mL afforded a high sensitivity and specificity in terms of HCC detection in FALD patients. The survival rate was lower in patients with HA levels ≥ 55.5 ng/mL. Thus, the HA level may serve as a useful marker of HCC development and FALD survival.

Using a formula to estimate the extent of fibrosis, Yoo et al. [26] found that the APRI score, T-BIL and albumin levels, and white blood cell count were significantly correlated with liver stiffness (evaluated via transient elastography) in Fontan patients. Notably, we found that FALD-HCC status did not significantly affect the APRI. Emamaullee et al. [13] reported that the APRI, MELD-XI score, FIB-4 index, and T-BIL level significantly increased as fibrosis progressed, while the platelet count decreased. However, the changes in the MELD and MELD-sodium scores were not statistically significant. The MELD-XI score was reportedly correlated with the fibrosis stage; the scores for patients with stages F3–4 and F0–2 were 11.6 ± 3.8 and 10.4 ± 2.1, respectively. We found that the FIB-4 index significantly increased and the MELD-XI score tended to increase in FALD-HCC patients as they developed HCC. However, approximately half of all FALD patients on anticoagulants were not evaluated in terms of the MELD score. We found that the MELD-XI scores increased in FALD-HCC patients; the score reliably detected high-risk patients. The MELD-XI threshold for HCC detection was 18.7. Berg et al. reported that patients with MELD-XI scores ≥ 19 had higher mortality rates than other patients [27]; our MELD-XI ≥ 18.7 threshold was similar to their findings. Munsterman et al. [28] found that the MELD-XI scores were low even in patients with liver fibrosis. A MELD-XI score ≥ 18.7 afforded high specificity but low sensitivity. The use of a MELD-XI cutoff of 12.2 increased the specificity to 0.733. Although the MELD-XI score was not a significant predictor of survival, Abe et al. [29] reported that the score accurately reflected the prognosis of patients with heart failure. We also found that the MELD-XI score was predictive of patient outcomes. In contrast, although the FIB-4 index increased (to 0.84) in FALD-HCC patients, this index was not significantly predictive of HCC development.

Multivariate Cox regression analysis indicated that the risk factors for HCC development were polysplenia and higher MELD-XI scores. Although, the mechanism for the increased HCC risk in polysplenia patients is unknown, HCC development has been reported in cases of situs ambiguus with polysplenia [30–32]. We hypothesized that polysplenia complicated by other abnormalities, such as visceral inversions or more severe cardiac abnormalities, could promote HCC development. The FIB-4 index has been reported as a risk factor for HCC [8]. In this study, however, we found that MELD-XI scores were more significant predictors for the risk of HCC development.

In terms of cardiac parameters, the BNP level is a significant predictor of cardiovascular events and death in patients with the atriopulmonary connection imparted by Fontan surgery [33]. However, we found no difference in the BNP level between patients with and without HCC. Furthermore, right cardiac catheterization was not correlated with the CVP, EF, or HCC development. We speculate that HCC development was associated with the interval between examinations, as well as specific aspects of liver function.

The long-term survival rate after the Fontan procedure has been explored [34]. In one national study [7], 5 (0.19%) of 2,700 cases died. One systematic review [35] of 28 studies (6,707 patients) with approximately 10 years of follow-up reported 1,000 deaths from cardiac factors [heart/Fontan failure (22%)], arrhythmia (16%), respiratory failure (15%), renal disease (12%), and thrombosis/bleeding (10%). In our current study, 10 cases died; the survival rate was thus 96.9% at 20 years after Fontan surgery. Moreover, only one patient died within 0–5 years of FALD diagnosis. Possner et al. [22] reported that the 1-year survival rate was 50% in Fontan patients with HCC; such patients exhibited significantly worse prognosis than other patients. HCC development was associated with mortality. Early HCC detection is essential. Our results suggest that fibrosis markers may also predict mortality.

The limitations of our study include its single-center design and the small numbers of HCC cases. Additionally, we did not analyze all patients who underwent the Fontan operation; we lacked data concerning patients who did not exhibit liver disease. Referrals from the cardiologists to the gastroenterology unit may have been associated with a selection bias. A multivariate analysis is required to evaluate the risk factors for HCC development; notably, the numbers of HCC cases are increasing. Cardiac catheterization was not performed for all cases; in some instances, catheterization (if performed) was not conducted at the time of HCC development.

## Conclusions

Of all FALD patients analyzed, 15 (9.7%) developed HCC within 20 years after the Fontan procedure. The HCC risk was higher in patients with cirrhosis, esophageal varices, or congenital polysplenia. The HA fibrosis marker and the T-BIL and MELD-XI markers of liver function were predictive of HCC progression and overall prognosis in FALD patients.
